# Construction of K_3_PW_12_O_40_/ZnIn_2_S_4_ heterostructures with a likely direct Z-scheme pathway for enhanced visible-light photocatalytic hydrogen evolution and antibiotic degradation

**DOI:** 10.1039/d6ra09625e

**Published:** 2026-11-09

**Authors:** Yong Chen, Xiaowei Wen, Chunhong Yu, Dawei Lu, Feng Li, Taohai Li

**Affiliations:** a School of New Energy, Nanjing University of Science and Technology Jiangyin 214000 Jiangsu China; b College of Chemistry, Key Lab of Environment Friendly Chemistry and Application in Ministry of Education, Xiangtan University Xiangtan 411105 Hunan China hnlth@xtu.edu.cn

## Abstract

Developing efficient visible-light-responsive photocatalysts for solar fuel production and pollutant remediation remains challenging because of insufficient light harvesting and rapid recombination of photogenerated charge carriers. Herein, K_3_PW_12_O_40_/ZnIn_2_S_4_ (KPW/ZIS) heterostructured composites were prepared *via* a facile solvothermal route and evaluated for visible-light-driven H_2_ evolution and tetracycline hydrochloride (TC-HCl) degradation. Structural and spectroscopic characterizations confirmed the integration of KPW with flower-like ZIS and the formation of intimate interfacial contact between the two components. The incorporation of ZIS broadened the visible-light response of KPW and promoted the separation and interfacial transfer of photogenerated charge carriers. Among the prepared composites, ZK-3 exhibited the highest photocatalytic activity, achieving an H_2_ evolution rate of 325.61 ± 5.22 µmol g^−1^ h^−1^ and a TC-HCl removal efficiency of 69.88 ± 0.91% within 120 min. The corresponding apparent pseudo-first-order rate constant for TC-HCl degradation was 0.010 min^−1^. Photoelectrochemical measurements, band-structure analysis, reactive-species trapping experiments, and electron spin resonance measurements collectively supported a possible direct Z-scheme charge-transfer pathway. In addition, ZK-3 retained its photocatalytic activity during repeated cycling tests. These results provide a viable approach to constructing polyoxometalate-based sulfide heterostructures for bifunctional solar-energy conversion and environmental remediation.

## Abstract 2

A K_3_PW_12_O_40_/ZnIn_2_S_4_ heterostructure was constructed *via* a solvothermal strategy, enabling enhanced visible-light-driven H_2_ evolution and tetracycline degradation through improved charge separation and a likely direct Z-scheme pathway.

## Graphical abstract

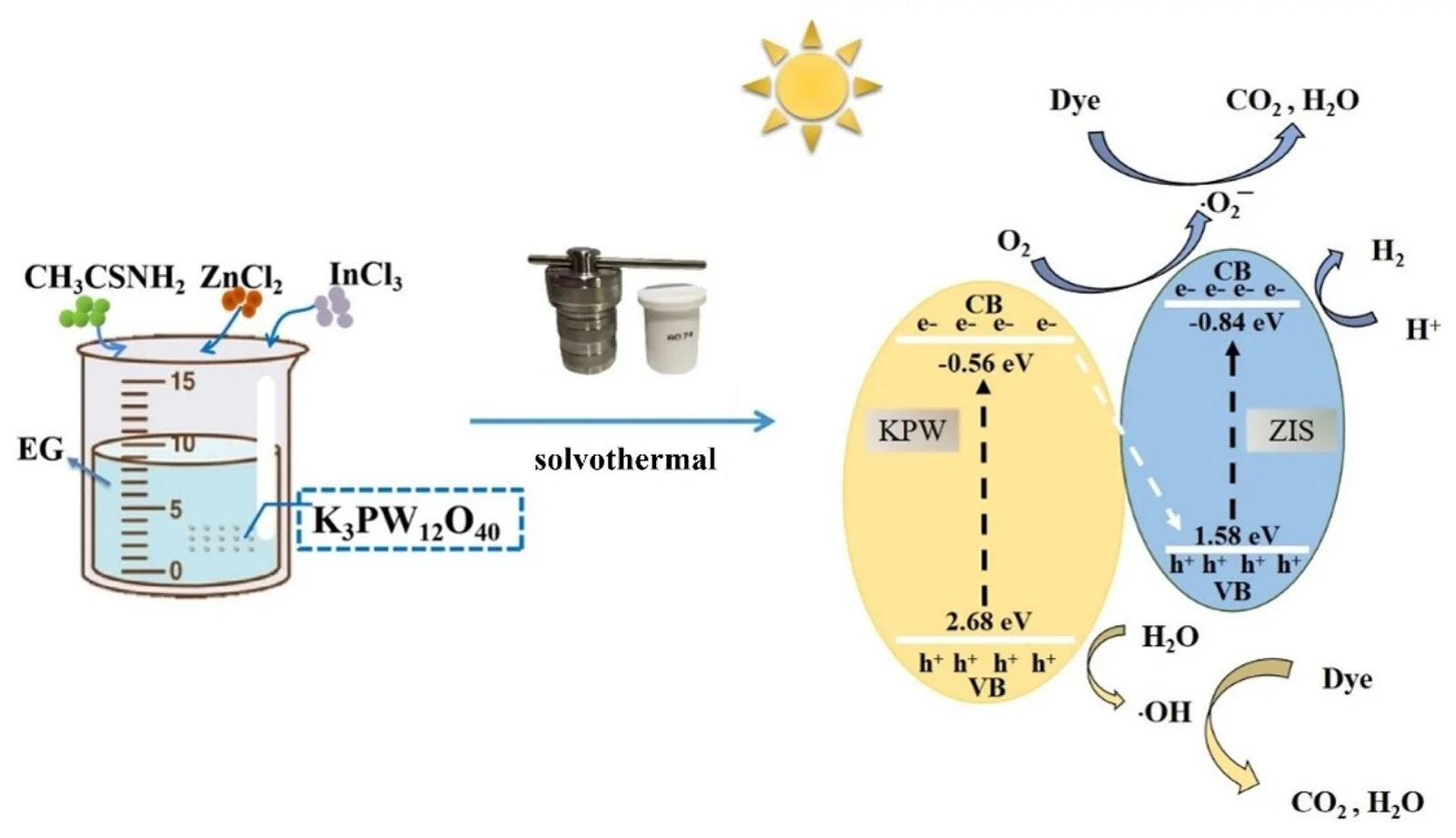

## 1. Introduction

Photocatalysis has attracted extensive attention as a promising approach for addressing both energy shortage and environmental pollution through solar energy utilization.^1–3^ Among various photocatalytic processes, solar-driven hydrogen evolution and degradation of refractory organic contaminants are particularly attractive because they enable simultaneous solar-to-chemical energy conversion and pollutant abatement.^4–7^ However, the practical performance of many semiconductor photocatalysts remains limited by insufficient visible-light harvesting, rapid recombination of photogenerated charge carriers, and sluggish surface reaction kinetics.^8,9^ Therefore, the rational design of efficient visible-light-responsive photocatalysts with enhanced charge separation and interfacial reaction efficiency remains an important research objective.

Polyoxometalates (POMs), a class of discrete and structurally well-defined metal–oxo clusters, have emerged as attractive functional materials because of their rich redox chemistry, reversible multi-electron transfer capability, and structural diversity.^10,11^ In particular, Keggin-type phosphotungstates have received considerable interest in photocatalysis owing to their high stability and strong electron-accepting ability.^12,13^ K_3_W_12_O_40_ (KPW), a potassium salt of the Keggin-type phosphotungstate anion, retains the intrinsic redox characteristics of the parent heteropolyanion and can exhibit greater structural robustness than the corresponding heteropolyacid under appropriate reaction conditions.^14,15^ Its electron-accepting ability may facilitate charge extraction from a coupled semiconductor, thereby suppressing the recombination of photogenerated electrons and holes. Nevertheless, the photocatalytic performance of pristine KPW is restricted by its limited visible-light absorption and inefficient charge-carrier separation.^16–18^ Coupling KPW with a visible-light-active semiconductor is therefore a viable strategy to overcome these drawbacks and improve its photocatalytic efficiency.

ZnIn_2_S_4_ (ZIS) is a layered ternary metal sulfide semiconductor widely investigated for photocatalytic H_2_ production and pollutant degradation because of its suitable band structure, visible-light response, and favorable reduction capability.^19–21^ In addition, ZIS can form hierarchical flower-like architectures assembled from thin nanosheets, which may expose abundant surface-active sites, shorten carrier-migration distances, and facilitate mass transfer.^22^ Despite these advantages, pristine ZIS still suffers from fast charge recombination and relatively weak oxidation ability, which limit its overall photocatalytic performance.^23^ Constructing a heterostructure between ZIS and a suitable redox-active component may therefore regulate interfacial charge migration and improve the utilization of photogenerated carriers.

Heterojunction engineering has been demonstrated to be an effective approach for enhancing photocatalytic activity by promoting directional charge migration and spatial charge separation across semiconductor interfaces.^24–26^ Compared with single-component photocatalysts, heterostructured systems often exhibit broader spectral response, faster interfacial charge transfer, and improved redox capability.^27,28^ In this context, integrating KPW with ZIS is particularly attractive because the two components possess complementary properties. ZIS, as a visible-light-active semiconductor, can serve as the main photosensitizing component and provide electrons with strong reducing power, whereas KPW can facilitate interfacial electron transport through its reversible multielectron redox behavior. Moreover, the nanosheet-assembled architecture of ZIS may provide abundant contact sites for anchoring KPW, thereby promoting the formation of intimate heterointerfaces. Such coupling is expected to improve charge separation and support both photocatalytic reduction and oxidation reactions. Although various bifunctional photocatalysts have been developed for H_2_ evolution and antibiotic degradation, many existing systems depend on noble-metal cocatalysts, involve complex synthetic processes, or exhibit photoactivity mainly under UV irradiation. Therefore, the development of noble-metal-free KPW/ZIS heterostructures through a facile synthesis strategy remains highly desirable, yet is still rarely explored, particularly for dual-function applications in visible-light-driven solar-fuel generation and antibiotic removal.

In this work, a series of KPW/ZIS heterostructured composites with different component ratios were prepared *via* a facile solvothermal route. Their phase composition, morphology, interfacial microstructure, surface chemical states, optical properties, and photoelectrochemical behavior were systematically investigated. The photocatalytic performance of the composites was evaluated through visible-light-driven H_2_ evolution and tetracycline hydrochloride (TC-HCl) degradation, with particular emphasis on the influence of ZIS content. The charge-carrier separation and transfer behavior was further examined using band-structure analysis, photoelectrochemical measurements, reactive-species trapping experiments, and electron spin resonance spectroscopy. On the basis of these results, a possible interfacial charge-transfer mechanism was proposed to explain the enhanced bifunctional photocatalytic performance of the KPW/ZIS heterostructure.

## 2 Results and discussion

### 2.1 Crystal structure and morphology

The crystal structures of the as-prepared samples were first characterized by X-ray diffraction (XRD). As shown in Fig. 1a, pristine KPW exhibits a series of sharp diffraction peaks, indicating its good crystallinity. These diffraction peaks can be well assigned to the characteristic crystalline phase of K_3_PW_12_O_40_ (PDF#72-9992), confirming the successful formation of the phosphotungstate salt.^29^ Pure ZIS shows diffraction peaks located at 21.6°, 27.7°, 30.4°, 39.8°, 47.2°, 52.4°, and 55.6°, corresponding to the (006), (102), (104), (108), (110), (116), and (022) crystal planes of hexagonal ZnIn_2_S_4_, respectively, in agreement with the standard card (PDF# 65-2023).^30–32^ For the KPW/ZIS composites, the characteristic peaks of KPW remain clearly visible, whereas the diffraction peaks of ZIS are relatively weak, likely owing to its lower content and weaker diffraction contribution in the composites. No obvious impurity peaks are detected, indicating that the solvothermal process does not induce additional crystalline phases. In addition, slight variations in peak position and relative intensity are observed after coupling with ZIS (Fig. 1b), implying interfacial interaction between the two components. These results confirm the successful construction of the KPW/ZIS composite system.

**Fig. 1:**
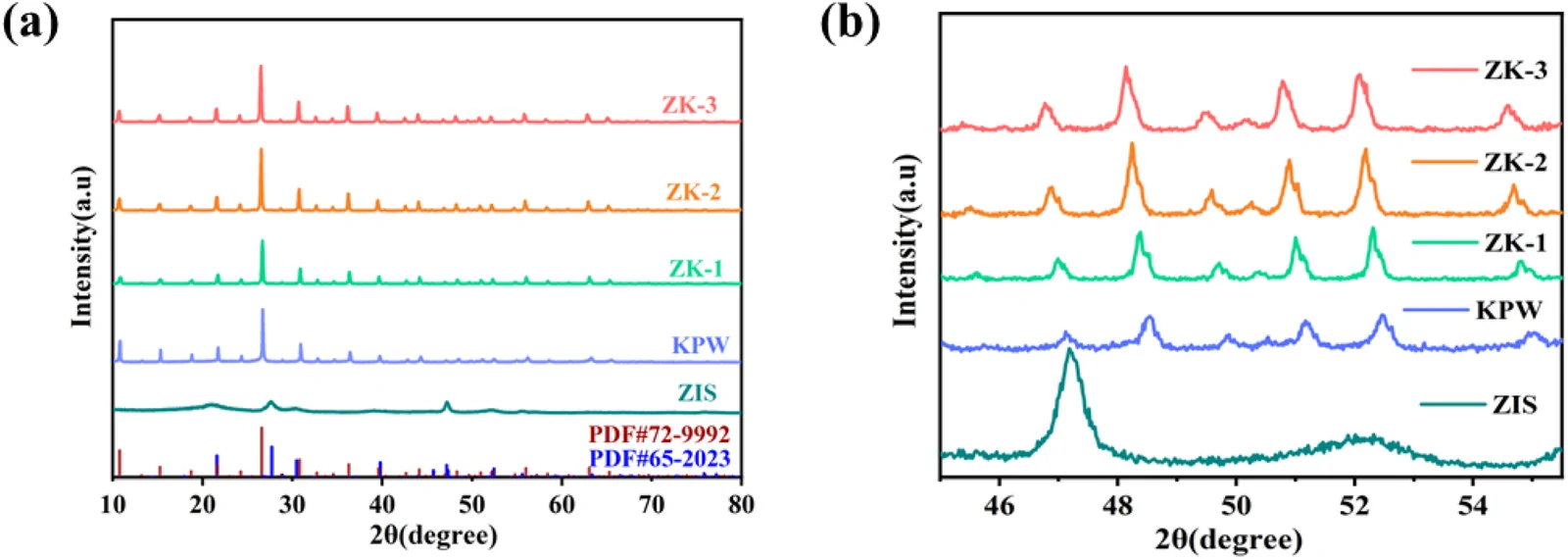
(a) XRD patterns of ZIS, KPW, and different KPW/ZIS composites; (b) enlarged XRD patterns of the selected diffraction region.

The morphology and microstructure of the samples were further examined by scanning electron microscopy (SEM) and transmission electron microscopy (TEM). As shown in Fig. 2a, pristine KPW mainly consists of relatively uniform polyhedral particles with micrometer-scale dimensions. A hollow interior can also be clearly observed, indicating the formation of a hollow polyhedral structure. Such an architecture is beneficial for enlarging the accessible surface area, facilitating mass transport, and enhancing light scattering. In contrast, pristine ZIS displays a typical flower-like microspherical morphology assembled from ultrathin nanosheets (Fig. 2b). This hierarchical structure provides abundant exposed active sites and short diffusion paths for photogenerated carriers. After solvothermal coupling, the composite sample retains the main morphological characteristics of both components. As shown in Fig. 2c and d, KPW particles are closely anchored onto the ZIS nanosheet framework, indicating the formation of an intimate heterointerface. This configuration is expected to increase the interfacial contact area and shorten the migration distance of photogenerated charge carriers. The TEM images in Fig. 2e and f provide further evidence of the composite microstructure. Distinct regions attributable to KPW and ZIS are in close contact, indicating the formation of an intimate heterointerface. Lattice fringes with interplanar spacings of 0.322 and 0.479 nm are observed, corresponding to the characteristic crystal planes of K_3_PW_12_O_40_ and hexagonal ZnIn_2_S_4_, respectively. The intimate interfacial coupling is expected to facilitate charge transfer during photocatalysis.

**Fig. 2:**
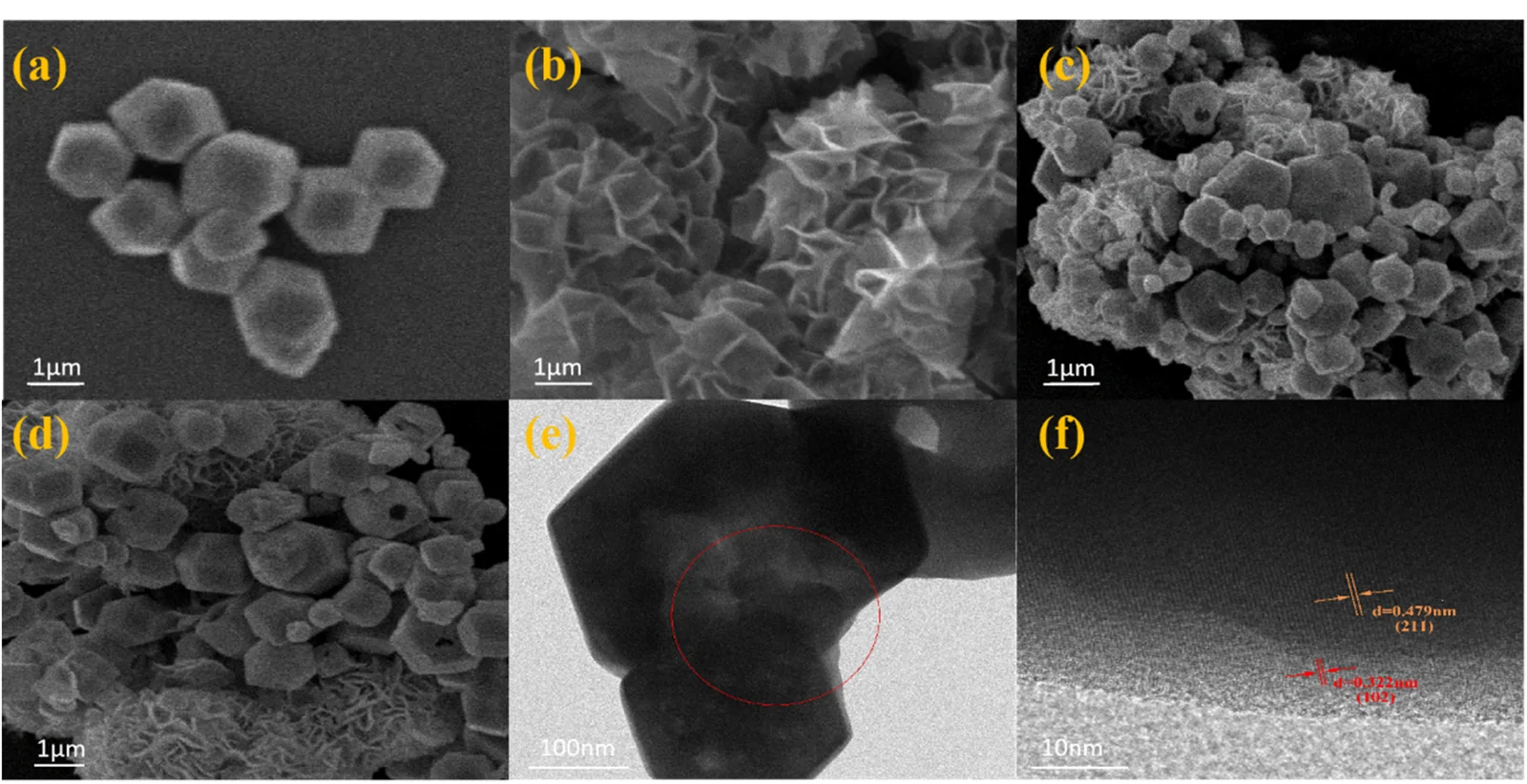
SEM images of (a) KPW, (b) ZIS, and (c and d) ZK-3; (e and f) TEM image of ZK-3.

To further elucidate the surface elemental composition and chemical states of the as-prepared samples, X-ray photoelectron spectroscopy (XPS) measurements were carried out. As shown in Fig. 3a, the survey spectrum of ZK-3 displays the characteristic signals of Zn, In, S, K, P, O, and W, confirming the coexistence of KPW and ZIS in the composite. In the high-resolution K 2p spectrum of ZK-3 (Fig. 3b), two peaks located at 293.0 and 295.8 eV are assigned to K 2p_3/2_ and K 2p_1/2_, respectively, indicating the presence of K^+^ species. In the W 4f spectrum (Fig. 3c), the two major peaks centered at 35.6 and 38.7 eV correspond to W 4f_7/2_ and W 4f_5/2_ of W(vi), respectively, consistent with the tungsten species in KPW. The O 1s spectrum (Fig. 3d) can be deconvoluted into two components, where the lower-binding-energy peak at 530.63 eV is attributed to lattice oxygen associated with O–W and O–P bonds, while the higher-binding-energy peak is assigned to surface-adsorbed oxygen-containing species such as hydroxyl groups or adsorbed water molecules.^33^ The P 2p peak at 134.07 eV (Fig. 3e) is characteristic of P(v),^34^ further confirming the structural integrity of the phosphotungstate structure.

**Fig. 3:**
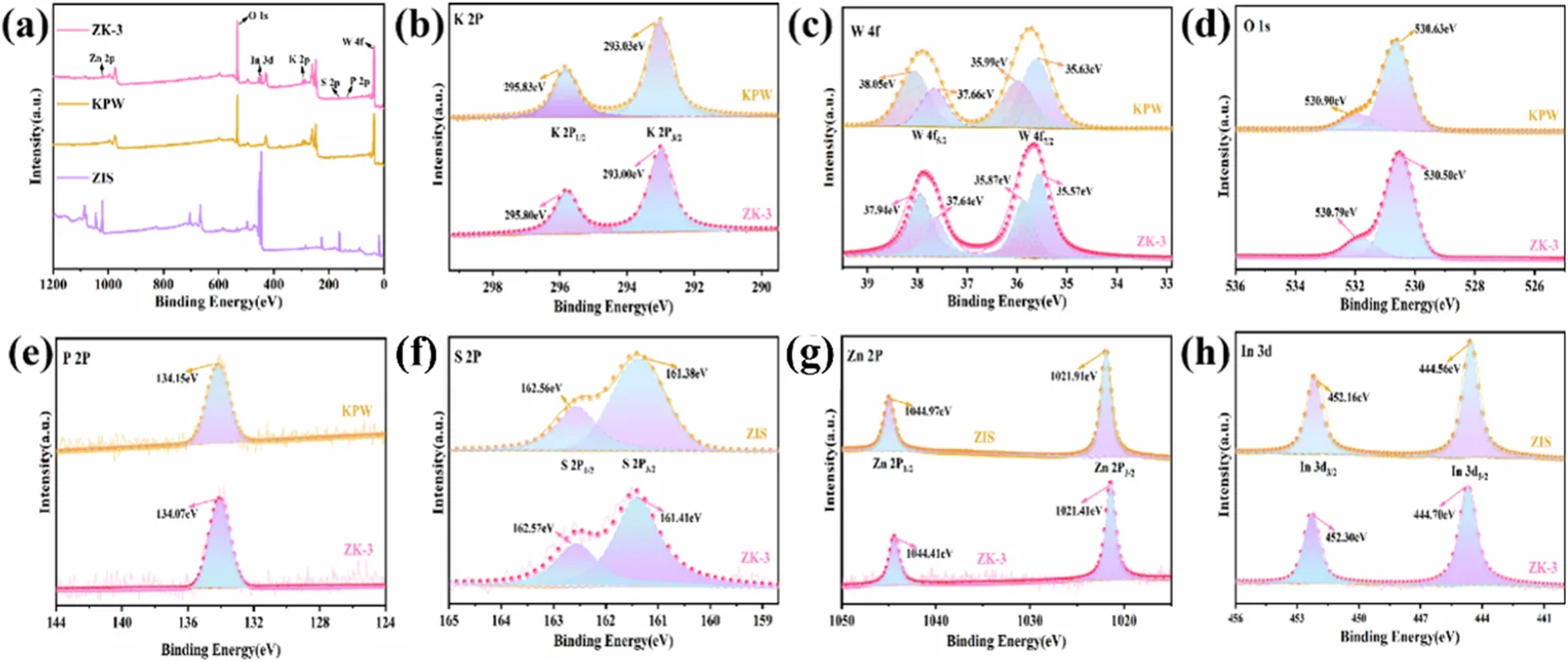
XPS spectra of ZK-3, KPW and ZIS samples: (a) survey spectrum; high-resolution spectra of (b) K 2p, (c) W 4f, (d) O 1s, (e) P 2p, (f) S 2p, (g) Zn 2p, and (h) In 3d.

The S 2p spectrum of ZIS (Fig. 3f) can be fitted into two peaks corresponding to S 2p_3/2_ and S 2p_1/2_ of S^2−^. Compared with pristine ZIS (161.38 and 162.56 eV), the S 2p peaks of ZK-3 are slightly shifted to 161.41 and 162.57 eV, respectively, implying a changed electronic environment of sulfur after heterostructure formation. The high-resolution Zn 2p spectrum (Fig. 3g) of pristine ZIS displays two peaks at 1021.91 and 1044.97 eV, which are assigned to Zn 2p_3/2_ and Zn 2p_1/2_, respectively. In ZK-3, these peaks shift to 1021.41 and 1044.41 eV. The corresponding spin–orbit splitting values for pristine ZIS and ZK-3 are 23.06 and 23.00 eV, respectively, which are consistent with the Zn^2+^ oxidation state.^35,36^ As shown in Fig. 3h, the In 3d spectrum exhibits two characteristic peaks at 444.70 and 452.30 eV, assigned to In 3d_5/2_ and In 3d_3/2_ of In^3+^.^37^ Relative to pristine KPW, the K 2p, W 4f, O 1s, and P 2p signals of ZK-3 exhibit variations in binding energy. Changes are also observed for the ZIS-related signals following composite formation. These variations indicate that the local electronic environments of both components are modified upon coupling and therefore support the presence of interfacial electronic interaction between KPW and ZIS. Overall, the XPS results verify the successful construction of the KPW/ZIS heterostructure and reveal appreciable interfacial electronic coupling, which is beneficial for charge separation and migration.

The optical properties of the samples were investigated by UV-vis diffuse reflectance spectroscopy (DRS). As shown in Fig. 4a, pristine KPW exhibits strong absorption in the ultraviolet region but negligible response in the visible region, with an absorption edge of approximately 390 nm. In contrast, pristine ZIS displays broad absorption in the visible region, with an absorption edge extending to around 520 nm, in agreement with previous reports.^38,39^ After coupling with ZIS, ZK-3 displays a clear red shift in the absorption edge relative to pristine KPW, together with significantly enhanced visible-light absorption. This observation indicates that the introduction of ZIS effectively broadens the spectral response range of KPW and improves the light-harvesting capability of the composite.

**Fig. 4:**
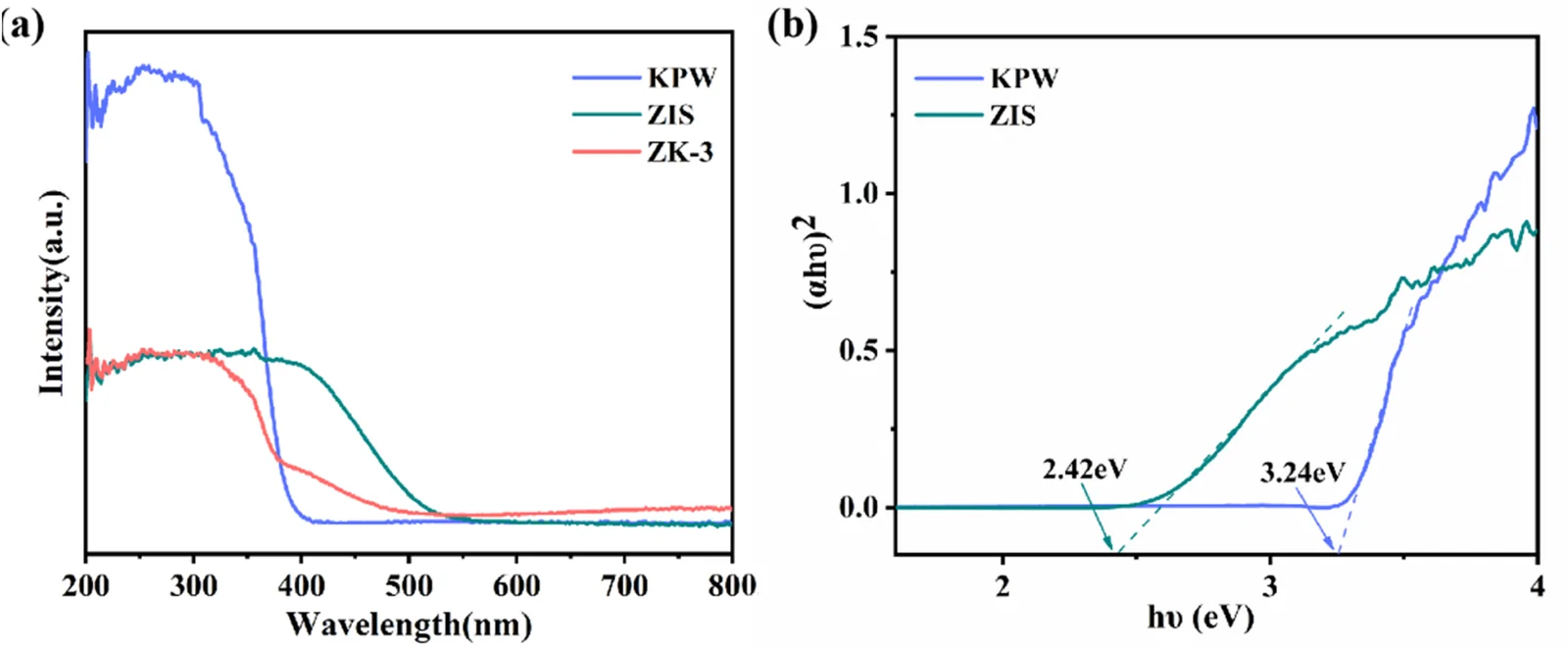
(a) UV-vis diffuse reflectance spectra of KPW, ZIS, and ZK-3; (b) corresponding Tauc plots for bandgap estimation.

To estimate the bandgap energies of the individual components, Tauc plots were constructed according to:

1 (*αhν*)^*n*^ = *A*(*hν* − *E*_g_)

where *α*, *hν*, *A*, and *E*_g_ represent the absorption coefficient, photon energy, proportionality constant, and bandgap energy, respectively. For direct allowed transitions, *n* = 2.^40^ Because KPW and ZIS have been reported as direct-bandgap semiconductors,^38,39^ their bandgap values were determined from the plots of (*αhν*)^2^*versus hν*, as shown in Fig. 4b. The calculated bandgap energies are 3.24 eV for KPW and 2.42 eV for ZIS, respectively, which agree well with previously reported values.^41^ These results indicate that coupling with ZIS markedly improves the visible-light response of the composite and provides a favorable basis for enhanced photocatalytic performance.

### 2.2 Photocatalytic H_2_ evolution

The photocatalytic H_2_ evolution performance of the as-prepared samples was subsequently evaluated under visible-light irradiation using lactic acid as a sacrificial reagent, without adding Pt as a cocatalyst. As shown in Fig. 5a and b, H_2_ generation increases progressively with irradiation time for all tested samples, while the activity strongly depends on the catalyst composition. Pristine KPW exhibits very low H_2_ evolution activity, affording only about 8 µmol of H_2_ under the tested conditions. Such limited performance can be attributed to its poor visible-light absorption and rapid recombination of photogenerated charge carriers, which severely restrict the effective utilization of photoinduced electrons and holes. After coupling with ZIS, the photocatalytic H_2_ evolution activity is markedly enhanced (Fig. 5b), demonstrating the beneficial effect of heterostructure construction. With increasing ZIS content, the photocatalytic activity first increases and then decreases. Among the prepared samples, ZK-3 delivers the highest H_2_ evolution rate of 325.61 ± 5.22 µmol g^−1^ h^−1^, which is approximately three times that of pristine KPW. This composition-dependent trend indicates that an appropriate KPW-to-ZIS ratio is essential for achieving an effective balance among visible-light absorption, heterointerfacial contact, surface-site accessibility, and charge-carrier migration. The decreased activity observed at higher ZIS contents may arise from excessive coverage of the KPW surface, a reduction in the proportion of effective heterointerfaces, or the introduction of additional charge-recombination sites. Because these possibilities cannot be distinguished solely from the activity measurements, further structural or kinetic evidence would be required to establish the dominant factor. Nevertheless, the superior activity of ZK-3 can reasonably be associated with the combined effects of improved visible-light harvesting and more efficient utilisation of photogenerated charge carriers at the KPW/ZIS interface.

**Fig. 5:**
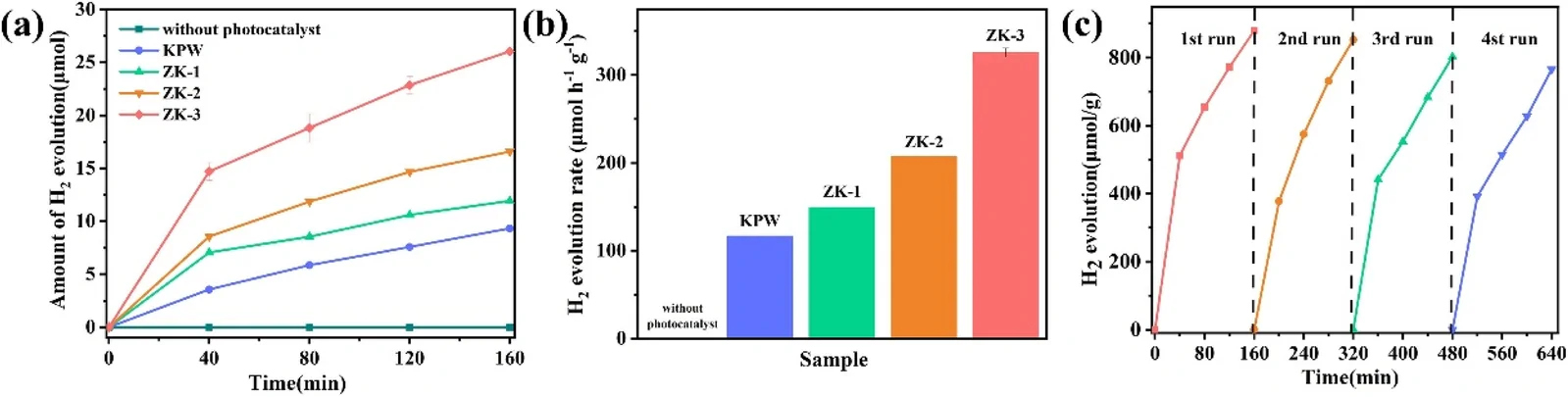
(a) Time-dependent photocatalytic H_2_ evolution over different samples under visible-light irradiation; (b) corresponding H_2_ evolution rates; (c) cycling stability of ZK-3 for photocatalytic H_2_ evolution.

To assess the stability of the photocatalyst, recycling tests were further carried out for ZK-3 under visible-light irradiation. As shown in Fig. 5c, no obvious decrease in the H_2_ evolution rate is observed after four consecutive cycles over a total reaction time of 640 min, demonstrating the good photochemical stability and recyclability of the composite during the photocatalytic process.

### 2.3 Photocatalytic degradation of TC-HCl

The photocatalytic degradation of TC-HCl was further investigated to evaluate the environmental remediation capability of the obtained materials. As shown in Fig. 6a, negligible degradation of TC-HCl is observed in the absence of photocatalyst, indicating that self-photolysis under visible-light irradiation can be neglected. In the presence of pristine KPW, a noticeable decrease in TC-HCl concentration occurs during the dark adsorption stage, suggesting that KPW possesses a certain adsorption capacity toward TC-HCl. However, its photocatalytic degradation activity under visible-light irradiation remains limited.

**Fig. 6:**
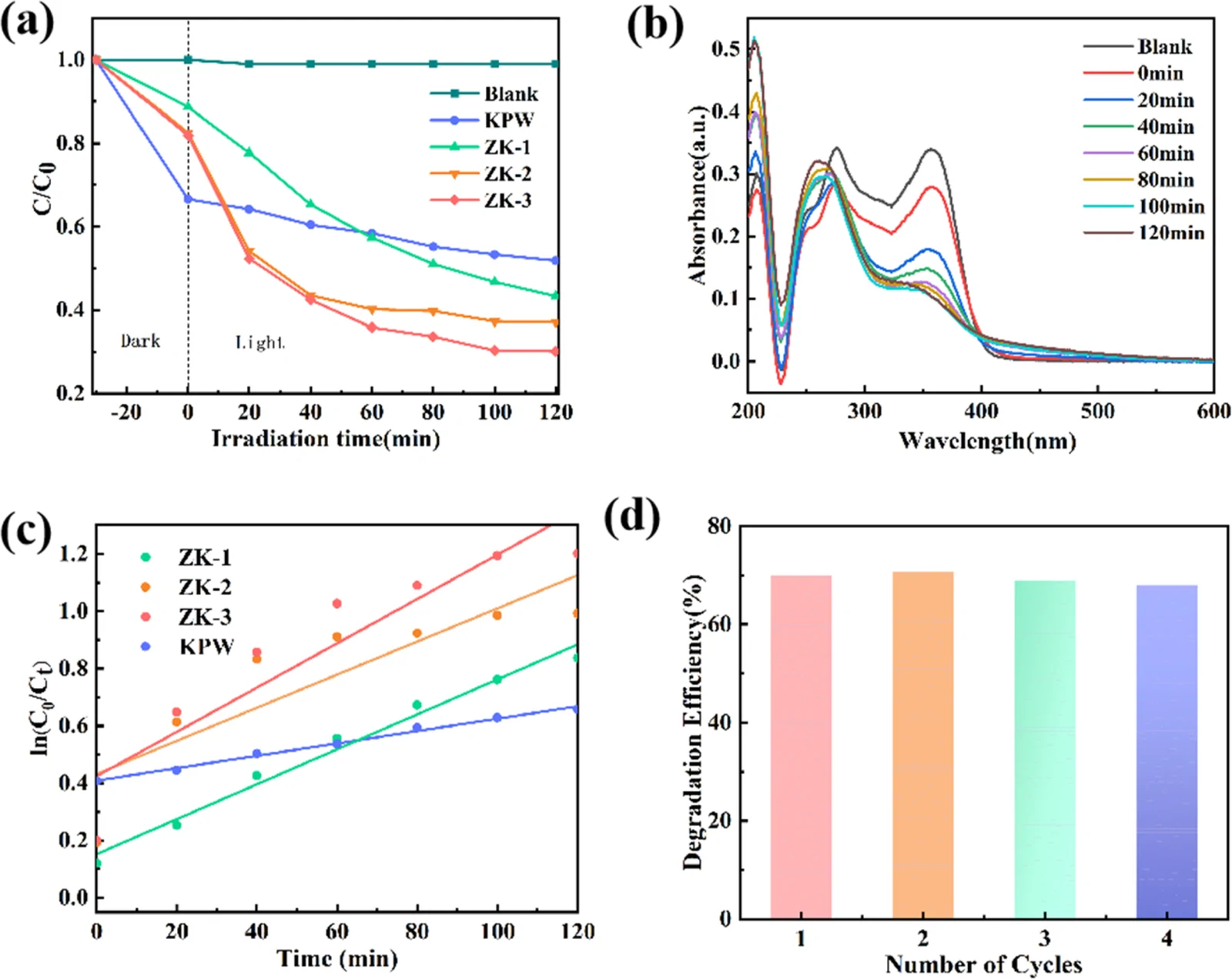
(a) Photocatalytic degradation of TC-HCl over different samples under visible-light irradiation; (b) time-dependent UV-vis absorption spectra of TC-HCl in the presence of ZK-3; (c) pseudo-first-order kinetic fitting curves for TC-HCl degradation; (d) cycling stability of ZK-3 for photocatalytic TC-HCl degradation.

By contrast, the KPW/ZIS composites exhibit significantly improved photocatalytic degradation performance, indicating that heterostructure formation effectively enhances the utilization of photogenerated charge carriers. The temporal evolution of the UV-vis absorption spectra of TC-HCl over ZK-3 under visible-light irradiation is shown in Fig. 6b. With increasing irradiation time, the characteristic absorption peak at around 360 nm gradually decreases, indicating the continuous degradation of TC-HCl molecules. Among all the tested samples, ZK-3 exhibits the highest photocatalytic degradation efficiency, reaching 69.88 ± 0.91% within 120 min. Together with its superior H_2_ evolution performance, this result demonstrates the bifunctional photocatalytic behavior of the optimized KPW/ZIS composite. The enhanced activity may be associated with improved visible-light harvesting, increased interfacial charge-transfer efficiency, and suppressed recombination of photogenerated carriers.

To quantitatively evaluate the degradation kinetics, the experimental data were fitted using a pseudo-first-order model. As shown in Fig. 6c, a good linear relationship is obtained for all samples, suggesting that the degradation of TC-HCl follows pseudo-first-order kinetics. The apparent reaction rate constant of ZK-3 is calculated to be 0.010 min^−1^, which is 2.2 times higher than that of pristine KPW. The kinetic results are consistent with the observed degradation efficiencies and further confirm the beneficial effect of coupling KPW with an appropriate amount of ZIS.

The recycling stability of ZK-3 was also assessed in the TC-HCl degradation system. As shown in Fig. 6d, after four consecutive cycles, the degradation efficiency remains at 68.03%, with only a slight decrease of 1.86% relative to the initial cycle. This result demonstrates that ZK-3 possesses good structural stability and reusability during photocatalytic pollutant degradation. The good stability is mainly associated with the robust KPW/ZIS interfacial coupling and the structural durability of both components.

It should be noted that the reported performance was obtained using a laboratory-scale single-contaminant system. In practical water matrices, variations in pH, dissolved inorganic ions, natural organic matter, and coexisting contaminants may alter the adsorption behavior, reactive-species generation, and degradation kinetics. Accordingly, the present results should be regarded as proof of concept for visible-light-driven antibiotic removal rather than direct evidence of practical wastewater-treatment efficiency.

### 2.4 Photoelectrochemical properties and mechanism analysis

To gain further insight into the separation and transfer of photogenerated charge carriers, photoluminescence (PL), transient photocurrent response, electrochemical impedance spectroscopy (EIS), and Mott–Schottky analyses were performed. PL emission usually originates from the radiative recombination of photogenerated charge carriers and is therefore widely used to evaluate charge separation efficiency in photocatalytic systems.^42^ As shown in Fig. 7a, under an excitation wavelength of 295 nm, the KPW/ZIS composites exhibit markedly lower PL intensities than pristine KPW, indicating suppressed recombination of photogenerated carriers after heterostructure formation. Among all the samples, ZK-3 shows the weakest PL emission, suggesting the highest charge separation efficiency, in agreement with its superior photocatalytic activity.

**Fig. 7:**
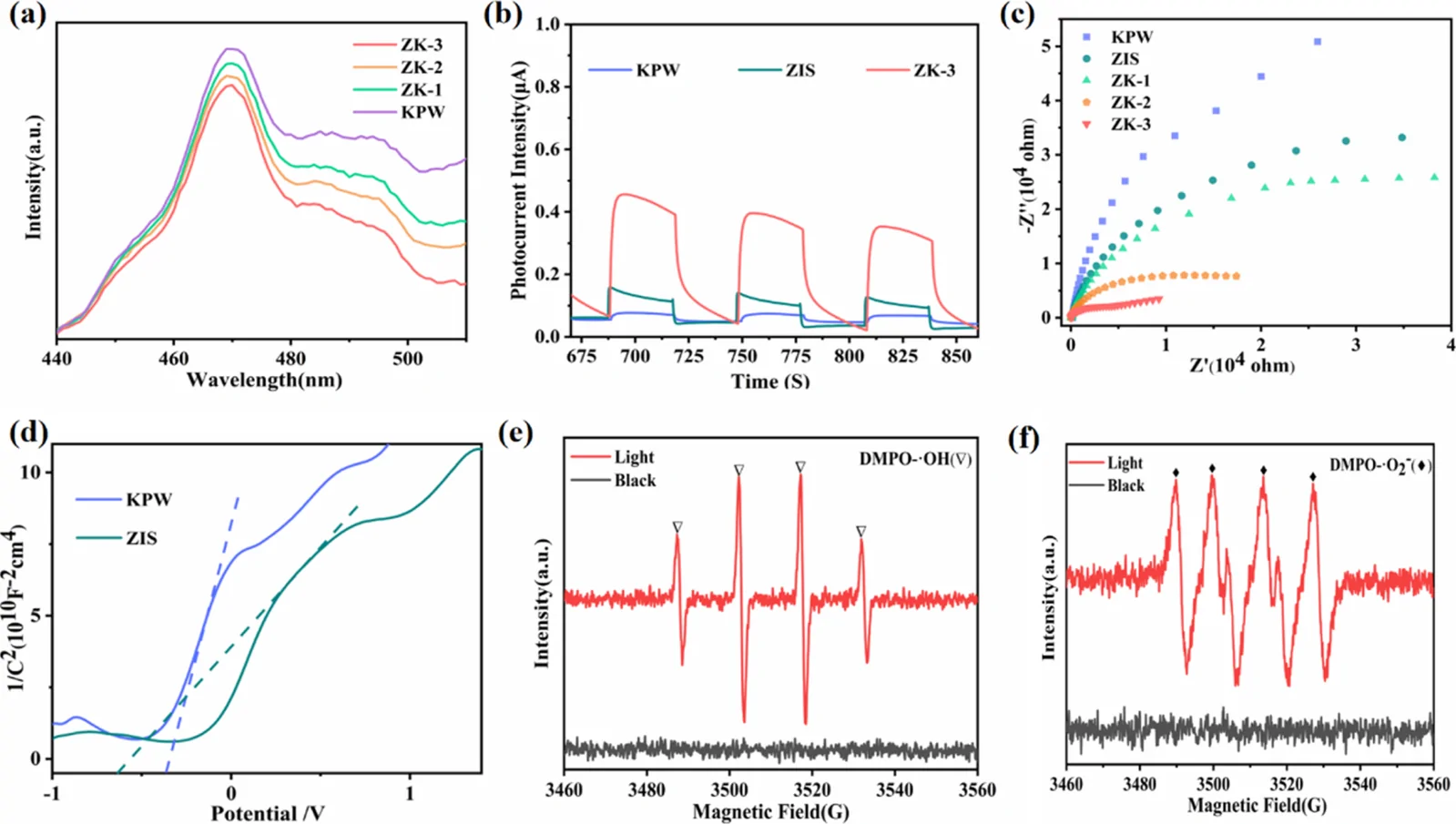
(a) PL spectra of KPW and KPW/ZIS samples; (b) transient photocurrent responses of KPW, ZIS, and ZK-3; (c) EIS Nyquist plots of KPW, ZIS, and KPW/ZIS samples; (d) Mott–Schottky plots of KPW and ZIS; (e and f) ESR spectra of ZK-3 for DMPO-˙O_2_^−^ and DMPO-˙OH under dark and visible-light conditions.

The transient photocurrent responses of the samples were recorded under intermittent visible-light irradiation. ZK-3 exhibits a higher and more reproducible photocurrent response than pristine KPW and ZIS (Fig. 7b), suggesting more efficient generation and migration of photoinduced carriers.^43^ In addition, ZK-3 shows a smaller semicircle in the electrochemical impedance spectroscopy Nyquist plot (Fig. 7c), indicating lower interfacial charge-transfer resistance. The combined PL, photocurrent, and EIS results demonstrate that coupling KPW with ZIS facilitates charge-carrier separation and interfacial transport, consistent with the improved photocatalytic performance.

To clarify the band structures of KPW and ZIS, Mott–Schottky measurements were further conducted. As shown in Fig. 7d, both samples exhibit positive slopes, confirming their n-type semiconductor characteristics. The flat-band potentials of KPW and ZIS are estimated to be approximately −0.36 and −0.64 V, respectively. For n-type semiconductors, the conduction band (CB) potential is generally close to, or slightly more negative than, the flat-band potential.^44,45^ Accordingly, the CB positions of KPW and ZIS are estimated to be around −0.56 and −0.84 eV, respectively. Combined with the optical bandgap values derived from UV-vis DRS, the valence band (VB) otentials can be calculated according to:

2 *E*_g_ = *E*_VB_ − *E*_CB_

Thus, the VB positions of KPW and ZIS are calculated to be 2.68 and 1.58 eV, respectively. The difference in band positions is favorable for constructing an effective interfacial charge-transfer pathway.

To identify the reactive species involved in the photocatalytic process, electron spin resonance (ESR) measurements were performed using ZK-3 as the representative sample. As shown in Fig. 7e and f, no obvious ESR signals are observed in the dark, whereas distinct signals assigned to DMPO-˙O_2_^−^ and DMPO-˙OH appear under visible-light irradiation, indicating the formation of both superoxide radicals and hydroxyl radicals during photocatalysis.^46,47^ These reactive species are expected to contribute substantially to the degradation of TC-HCl.

Based on the above results, a possible charge-transfer mechanism is proposed for the KPW/ZIS heterostructure. If a conventional type-II heterojunction were formed, electrons would migrate from the CB of ZIS to the CB of KPW, while holes would transfer from the VB of KPW to the VB of ZIS. Although this process could facilitate spatial charge separation, the holes remaining on the VB of ZIS would possess relatively weak oxidation ability, which does not satisfactorily account for the experimentally observed generation of ˙OH radicals. By contrast, a likely direct Z-scheme charge-transfer pathway can better account for both the improved carrier separation and the preservation of strong redox ability. In this mechanism, photogenerated electrons in the CB of KPW recombine with holes in the VB of ZIS at the interface, while the electrons retained in the CB of ZIS and the holes retained in the VB of KPW participate in the surface reduction and oxidation reactions, respectively (Fig. 8).

**Fig. 8:**
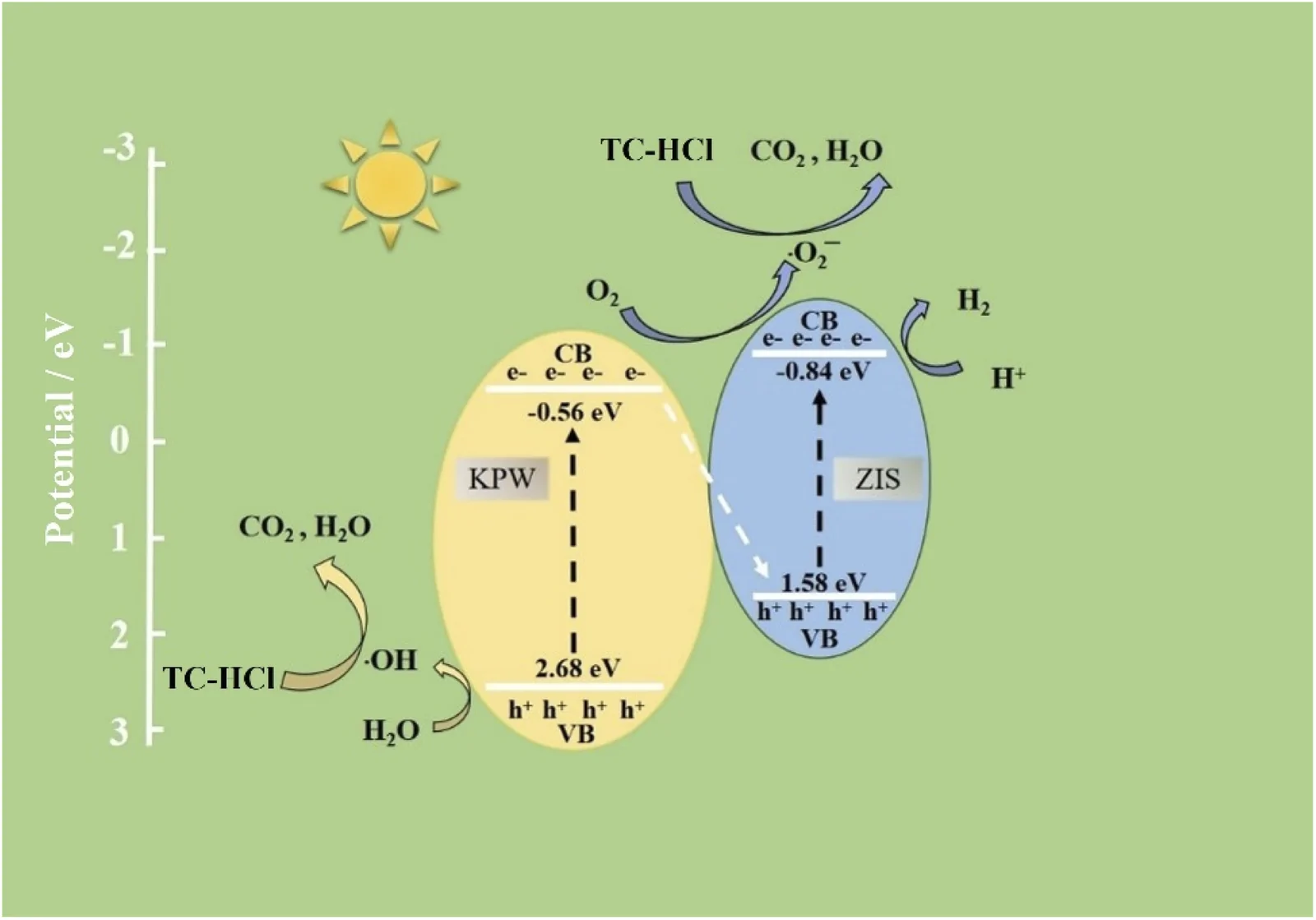
Schematic illustration of the likely direct Z-scheme charge-transfer pathway in the KPW/ZIS heterostructure under visible-light irradiation.

Under visible-light irradiation, ZIS is readily excited to generate electrons and holes, while KPW participates in the interfacial photoinduced charge-transfer process. The electrons accumulated on the CB of ZIS can reduce H^+^ to H_2_ in the H_2_ evolution system or react with dissolved O_2_ to produce ˙O_2_^−^ in the degradation system. Meanwhile, the holes preserved on the VB of KPW possess stronger oxidation ability and can directly oxidize organic molecules or promote the formation of ˙OH radicals, thereby facilitating TC-HCl degradation. Therefore, the improved bifunctional photocatalytic activity of the composite can be mainly attributed to the synergistic effects of enhanced visible-light harvesting, intimate interfacial contact, and more effective charge separation through a likely Z-scheme pathway.

## 3. Conclusions

In summary, a series of KPW/ZIS heterostructured composites were prepared *via* a solvothermal route for visible-light-driven H_2_ evolution and TC-HCl degradation. Structural and microscopic characterizations confirmed the coexistence of KPW and ZIS and revealed intimate interfacial contact between the two components. The incorporation of flower-like ZIS broadened the visible-light response of KPW, while interfacial coupling promoted the separation and transfer of photogenerated charge carriers. Among the prepared composites, ZK-3 exhibited the highest photocatalytic activity, achieving an H_2_ evolution rate of 325.61 ± 5.22 µmol g^−1^ h^−1^ and a TC-HCl removal efficiency of 69.88 ± 0.91% within 120 min. The apparent pseudo-first-order rate constant for TC-HCl degradation reached 0.010 min^−1^. In addition, ZK-3 maintained stable photocatalytic performance during repeated cycling experiments. Band-structure analysis, photoelectrochemical measurements, reactive-species trapping experiments, and ESR spectroscopy collectively supported a possible direct Z-scheme charge-transfer pathway. The enhanced activity was attributed to the combined effects of improved visible-light harvesting, intimate interfacial contact, and more efficient utilisation of photogenerated charge carriers. This study demonstrates the potential of polyoxometalate–sulfide heterostructures as noble-metal-free bifunctional photocatalysts for solar-fuel production and contaminant removal.

## 4. Experimental

### 4.1 Materials

Phosphotungstic acid hydrate (H_3_PW_12_O_40_·*x*H_2_O), potassium nitrate (KNO_3_), thioacetamide (C_2_H_5_NS), zinc chloride (ZnCl_2_), indium chloride (InCl_3_), ethylene glycol (C_2_H_6_O_2_), lactic acid, and tetracycline hydrochloride (TC-HCl) were of analytical grade and used as received without further purification. Deionized water was used throughout all experiments.

### 4.2 Preparation of K_3_PW_12_O_40_(KPW)

K_3_PW_12_O_40_ (KPW) was prepared by a coprecipitation method. H_3_PW_12_O_40_·*x*H_2_O (15.00 g) was dissolved in 200 mL of deionized water, while KNO_3_ (3.16 g) was dissolved in 100 mL of deionized water. The KNO_3_ solution was then added dropwise to the H_3_PW_12_O_40_ solution under continuous stirring, resulting in the gradual formation of a white precipitate. After further stirring for 30 min, the precipitate was collected, washed alternately with water and ethanol three times, and dried under vacuum at 60 °C for 12 h. The obtained solid was ground into a fine powder and denoted as KPW.

### 4.3 Preparation of K_3_PW_12_O_40_/ZnIn_2_S_4_ composite (KPW/ZIS)

The KPW/ZIS composites were prepared *via* a solvothermal route. In a typical synthesis, 3.00 g of KPW (1 mmol) was dispersed in 15 mL of ethylene glycol by ultrasonication. Then, ZnCl_2_ (0.042 g, 0.3 mmol) and InCl_3_ (0.14 g, 0.6 mmol) were added to the above suspension, followed by magnetic stirring for 30 min. Subsequently, 0.091 g of thioacetamide (1.2 mmol) was dissolved in 10 mL of deionized water and added dropwise to the mixture under continuous stirring. The resulting suspension was further ultrasonicated for 30 min to ensure uniform dispersion. After that, the mixture was transferred into a 50 mL Teflon-lined stainless-steel autoclave and heated at 180 °C for 12 h. After naturally cooling to room temperature, the product was collected by centrifugation, washed alternately with deionized water and ethanol three times, and dried at 70 °C. The dried sample was then ground into a fine powder for further use.

The composite prepared with a theoretical ZIS/KPW molar ratio of 0.3 : 1 was denoted as ZK-3. For comparison, additional composites with ZIS/KPW molar ratios of 0.1 : 1 and 0.2 : 1 were synthesized by proportionally adjusting the amounts of ZnCl_2_, InCl_3_, and thioacetamide, and the obtained samples were labeled ZK-1 and ZK-2, respectively. Pure ZIS was synthesized under the same solvothermal conditions in the absence of KPW and denoted as ZIS.

### 4.4 Characterization

Powder X-ray diffraction (XRD, MiniFlex II) with Cu Kα radiation (*λ* = 0.15406 nm) was used to determine the crystal structures of the samples. X-ray photoelectron spectroscopy (XPS, ESCA Lab MKII) was employed to analyze the elemental composition and surface chemical states. The C 1s core level at 284.6 eV was taken as an internal reference to correct the shift of the binding energies. The morphology and microstructure were characterized by field-emission scanning electron microscopy (FESEM, JEOL JSM-6700F) and transmission electron microscopy (TEM, JEM-2100F). UV-vis diffuse reflectance spectra (DRS) were recorded on a Lambda-25 spectrophotometer using BaSO_4_ as the reference. Photoluminescence (PL) spectra were collected on an LS55 fluorescence spectrometer with an excitation wavelength of 295 nm. Photoelectrochemical measurements were carried out on a CHI660E electrochemical workstation using a conventional three-electrode system. The prepared sample-coated electrode, a platinum wire, and an Ag/AgCl electrode were used as the working, counter, and reference electrodes, respectively. Electrochemical impedance spectroscopy (EIS) and transient photocurrent response were measured under visible-light irradiation.

### 4.5 Photocatalytic hydrogen evolution

Photocatalytic H_2_ evolution was evaluated in real time using an online photocatalytic analysis system (LabSolar-III AG, Beijing Perfectlight, China) under irradiation from a 300 W Xe lamp (PLS-SXE300+, Beijing Perfectlight, China) with a UV-cutoff filter (*λ* > 420 nm). In a typical run, 30 mg of photocatalyst was dispersed in 100 mL of aqueous solution containing 10 vol% lactic acid as a sacrificial reagent. Before irradiation, the suspension was degassed and purged with N_2_ for 30 min to remove dissolved air and establish an inert atmosphere. During the photocatalytic reaction, the suspension was continuously stirred to maintain uniform dispersion. The amount of evolved H_2_ was quantified at given time intervals using a gas chromatograph (GC-6890A, Zhongkehuifen, China) equipped with a thermal conductivity detector (TCD) with argon as the carrier gas. The final photocatalytic hydrogen evolution activity of the optimal sample is obtained from the mean of three independent replicate experiments, and standard deviations are provided for all data points.

### 4.6 Photocatalytic degradation of TC-HCl

Photocatalytic degradation experiments were performed using tetracycline hydrochloride (TC-HCl) as a model pollutant. Typically, 50 mg of photocatalyst was dispersed in 50 mL of TC-HCl aqueous solution (10 mg L^−1^). Prior to light irradiation, the suspension was magnetically stirred in the dark for 30 min to establish adsorption–desorption equilibrium. Subsequently, the suspension was exposed to visible light under continuous stirring. At predetermined time intervals, aliquots were withdrawn, centrifuged to remove the catalyst particles, and then analyzed by UV-vis spectroscopy to determine the residual concentration of TC-HCl.

The degradation efficiency was calculated according to:
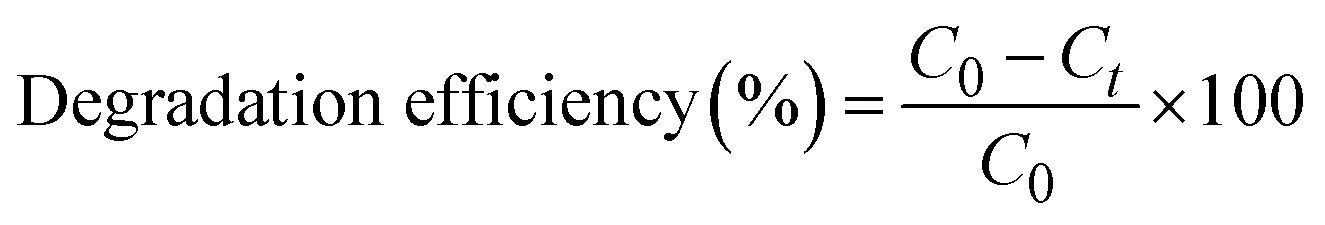
where *C*_0_ is the concentration of TC-HCl after dark adsorption equilibrium and *C*_*t*_ is the concentration at irradiation time *t*. The final photocatalytic TC-HCl degradation activity of the optimal sample is obtained from the mean of three independent replicate experiments, and standard deviations are provided for all data points.

For kinetic analysis, the degradation data were fitted using a pseudo-first-order model:
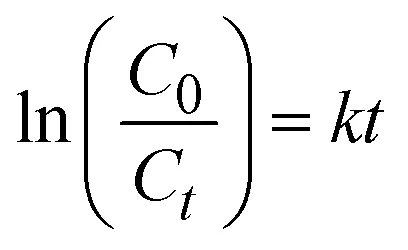
where *k* is the apparent reaction rate constant.

## Author contributions

Yong Chen: writing, investigation, writing – review & editing, funding acquisition. Xiaowei Wen: writing, investigation, formal analysis, visualization. Chunhong Yu: investigation, formal analysis, visualization, validation. Dawei Lu: investigation, formal analysis, visualization. Feng Li: conceptualization, funding acquisition. Writing – review & editing, data curation. Taohai Li: methodology, supervision, data curation, project administration. All authors have given approval to the final version of the manuscript.

## Conflicts of interest

There are no conflicts to declare.

## Data Availability

The datasets used and analyzed during the current study are available from the corresponding author upon reasonable request.
